# Are Countries Becoming Better at SARS-CoV-2 Genomic Surveillance?

**DOI:** 10.3389/fpubh.2022.887955

**Published:** 2022-04-25

**Authors:** Utkarsha Mahanta, Gayatri Saberwal, Gaurav Sharma

**Affiliations:** Institute of Bioinformatics and Applied Biotechnology (IBAB), Bengaluru, India

**Keywords:** genome surveillance, SARS-CoV-2, public health, genome sequencing, GISAID

During 2020, all countries were, *inter alia*, refining their strategies in terms of genomic surveillance of the SARS-CoV-2 virus to keep their populations safe. After a year, each country must have become more efficient at this. To test this hypothesis, this study evaluates the genomic surveillance efforts of all countries in 2021 compared to 2020. We find that most countries are (i) doing more SARS-CoV-2 sequencing and (ii) submitting to GISAID faster in 2021. However, this improvement is not always adequate, given the increase in COVID-19 cases. One noteworthy change is that most developing countries have improved their genomic surveillance significantly. Therefore, this study recommends that all countries must (i) increase their sequencing efficiency, i.e., the number of sequenced genomes per 1,000 cases, and (ii) submit these sequences to platforms such as GISAID rapidly to keep up with the emergence of newer SARS-CoV-2 variants.

The COVID-19 outbreak that originated in Wuhan, China, in December 2019 was pronounced a pandemic on March 11, 2020 (1, 2). Although various vaccines are being used to stall the virus, the end is not in sight for most people. After 370 million cases, 5.7 million deaths, and two long, never-ending years, the emerging waves of evolved SARS-CoV-2 variants are still frightening.

In an unprecedentedly focused effort, for the past 2 years, researchers have worked hard to understand the biology and epidemiology of SARS-CoV-2. The availability of ~220,000 publications [identified by using the search terms “(SARS-CoV-2) OR (COVID-19)” in PubMed as of January 25, 2022] and ~7.5 million genome sequences in the international open-access public database (GISAID) is proof of the dedication of researchers to track, trace, and understand this virus and its associated disease. Starting from the sequence submission of the first SARS-CoV-2 genome on January 10, 2020, this global health emergency has led to extensive genomic surveillance in every corner of the world. This has led to the identification of multiple variants of concern such as Alpha, Beta, Gamma, Delta, Omicron, etc.; several variants of interest; and other variants that are being monitored (https://www.who.int/en/activities/tracking-SARS-CoV-2-variants/). However, two critical concerns relate to whether we are doing enough genomic surveillance and are we getting better at it with each passing year (3). In a previous work (4), we have suggested that the rapid submission of sequences to open-source portals such as GISAID is of utmost importance, along with increasing genome sequencing. Here, we have asked the following two questions, i.e., (i) have the number of SARS-CoV-2 sequenced genomes per thousand COVID-19 cases (sequencing efficiency) increased with time, and (ii) has the sample “Collection to Genome Submission Time Lag” (CSTlag) decreased with time? Based on these two parameters, we have evaluated how each country performed in 2021 compared to 2020.

To answer these questions, we used the latest GISAID data (raw data: 7,375,848 sequences; filtered and working data: 7,170,321), downloaded on January 25, 2022, to (i) identify the number of submitted SARS-CoV-2 genomes and (ii) the collection and genome submission dates for each sample. We classified the data into 2020 (634,211 samples) and 2021 (6,536,110 samples) based on the collection dates of each sample. We wanted to categorize all data in two broad categories (2020 and 2021); therefore, genomes collected and submitted in January 2022 were assigned to the latter category, i.e., 2021. We then calculated the CSTlag per sample, followed by the median CSTlag for each country in 2020 and 2021. Data for the number of COVID-19 cases per country was obtained from the open-access “Our World in Data” website (https://ourworldindata.org/covid-cases) on January 25, 2022. Based on this information, we calculated the fold-change in CSTlag (2020/2021) and sequencing efficiency (2021/2020), followed by plotting both values using log transformation (Figure 1).

**Figure 1:**
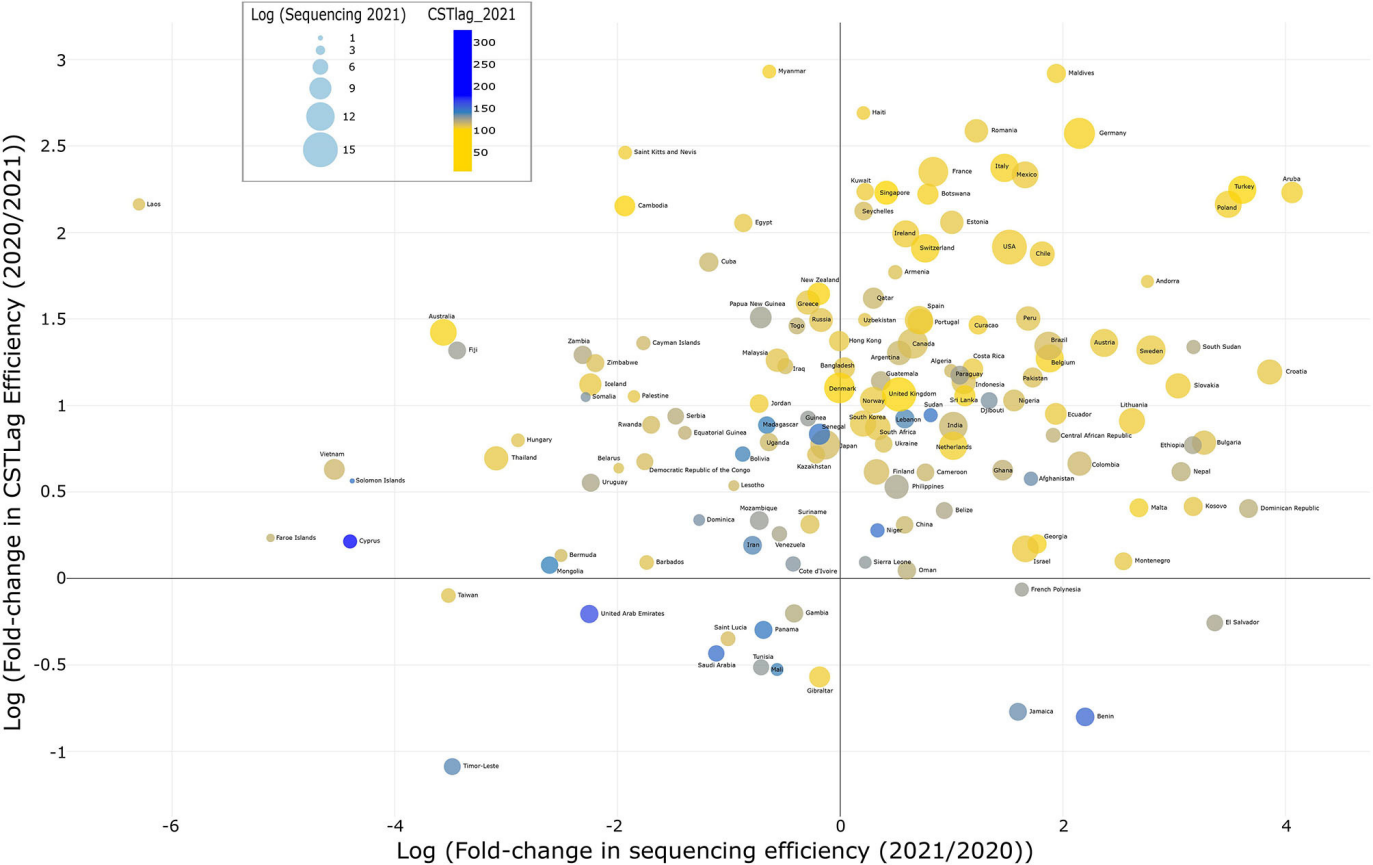
Mapping genomic surveillance efficiency: This bubble plot illustrates selected countries' comparative genome surveillance efficiency based on (i) sequencing efficiency and (ii) CSTlag efficiency in 2021 compared to 2020. The color and size of the bubble depict the median CSTlag per country and the number of submitted genomes in 2021, respectively. An interactive version, showing all countries' comparative genome surveillance efficiency, can be downloaded using this link https://drive.google.com/file/d/17TZ2RRgY84xlnbuFuGueokrqmThWZ5Jp/view?usp=sharing and visualized in an internet browser.

Based on our analysis of the genome-associated metadata of ~7 million SARS-CoV-2 samples submitted by researchers in 208 countries (Figure 1, Supplementary Table S1), we determined that, on a median scale, CSTlag efficiency has increased 4.5-fold (~78% decrease) within one year, i.e., from a median time of 85 days in 2020 to 19 days in 2021. This means that each sample, on average, was sequenced and submitted to GISAID 78% more rapidly in 2021 than in 2020. This is significant progress, which must be applauded. Among the six major continents (Supplementary Table S2), South American countries increased their sequencing efficiency 5.2-fold (from 1 to 5 per 1 k cases) and CSTlag efficiency 4.5-fold, whereas Oceania countries decreased by 95% (from 419 to 21 sequences per 1 k cases). In addition, Asia, Europe, and North America improved sequencing efficiency 2.0, 2.8, and 4.2 fold, whereas, with their continuous efforts (5, 6), Africa was able to improve 1.3-fold. Overall, most countries are persistently trying their best to stay ahead in this viral arms race (7).

Irrespective of the total of 7 million submitted sequences, only 90 out of 208 countries have sequenced at least 1,000 genomes since the pandemic; among these, the CSTlag efficiency ranges from −77% (Gibraltar) to 97% (French Guiana). To be noted, this study is focused on calculating the efficiency or improvement per country and must not be confused with the CSTlag values *per se*. For example, we identified that Gibraltar took a median of 13 days for 608 genome submissions in 2020 but a median of 23 days for 2,421 genomes in 2021, i.e., with a 77% decrease in sequencing efficiency. Saudi Arabia took a median of 128 days for 968 genome submissions in 2020 but 198 days for just 254 genomes in 2021, i.e., with a ~55% decrease in sequencing efficiency. Saudi Arabia is doing worse than Gibraltar regarding CSTlag values in 2020 and 2021 but has improved its sequencing efficiency compared to Gibraltar. Among the countries doing the most genomic surveillance, the USA has improved its CSTlag from 156 to 23 (6.7-fold), the UK from 26 to 9 days (2.9), and Germany from 196 to 15 days (13). Based on the submitted genomes, India is at 10th place and has improved its CSTlag from 159 in 2020 to 66 days in 2021 with a 2.5-fold change or 58% decrease.

The second criterion, i.e., sequencing efficiency, captures the genomic surveillance per 1000 COVID-19 cases. This study found that across the 90 countries that have submitted >1000 genomes, the sequencing efficiency ranges from 0.01 to 57-fold (−99 to 5,600%). There are several countries such as Aruba, Bahrain, Bosnia and Herzegovina, Botswana, Bulgaria, Cambodia, Costa Rica, Croatia, Cuba, Curacao, Ecuador, Georgia, Ghana, Guatemala, Lebanon, Nepal, Pakistan, Papua New Guinea, Slovakia, Sri Lanka, Trinidad and Tobago, and Vietnam, which did not perform much genomic surveillance in 2020 (<300 genomes); however, in 2021, they emerged as leaders with >1,000 genome submissions. In 2020, the USA was sequencing merely nine out of 1K cases; however, in 2021, it sequenced 43, a 4.6-fold change. The total number of cases in the UK increased 5.4-fold in 2021, whereas it raised its sequencing efficiency from 72 to 122 genomes per 1k cases (1.7-fold), demonstrating a 9-fold change in sequencing. Similarly, in Europe, Germany improved its sequencing efficiency from 5.6 to 49 per 1 k cases, France from 5.7 to 13, and Sweden from 6 to 103. Notably, Croatia had a 3-fold increase in cases, but it increased its sequencing 150-fold, increasing its sequencing efficiency from 0.6 to 27 genomes per 1 k cases (47-fold). Unlike most European countries, Iceland decreased sequencing efficiency by 90% (from 899 to 95 genome submissions per 1 k cases). In South America, Colombia enhanced its sequencing efficiency from 0.3 to 3 per 1 k cases (8.5-fold), Ecuador from 1.2 to 8.2 (6.9), Brazil from 0.9 to 5.6 (6.5), Chile from 2 to 13 (6.1), and Peru from 1.3 to 6.8 (5.4). Uruguay significantly dropped its sequencing efficiency from 9 per 1 k cases in 2020 to 1 in 2021. In Africa, Ghana and Nigeria improved their sequencing efficiency from 4.5 to 20 per 1 k cases (a 4.5-fold boost), whereas South Africa's sequencing efficiency was enhanced only 1.4-fold (6.1 to 8.6 per 1 k cases). In Asia, the cases in Turkey increased 4-fold, but it improved its genomic surveillance substantially with a 146-fold increase in sequencing and a 37-fold increase in sequencing efficiency, from 0.25 per 1 k cases in 2020 to 9.3 in 2021. Other countries such as Nepal, Maldives, Georgia, Pakistan, Bahrain, and Israel also improved significantly with a >5-fold change in sequencing efficiency. India and Indonesia also improved their SARS-CoV-2 surveillance from 1.2 to 3.5 per 1 k cases, whereas Singapore went from 27 to 41. The number of cases in the UAE increased 3-fold; however, its sequencing went down 0.3-fold, and CSTlag also decreased 0.8-fold (209 days to 257 days on the median scale). Notably, cases in Thailand increased 345-fold in 2021, whereas sequencing increased only 15-fold.

In 2020, all countries were evolving strategies to improve their genomic surveillance, providing medical aid and affordable vaccines, and overall, trying to keep their population safe and healthy. In 2021, each country was trying to build better infrastructure to sustain new variants' arrival and detect them as early as possible. The emergence of new variants of concern and variants of interest, enhanced transmission, improved scientific awareness, better funding (especially in developing countries), and various technological improvements in standardization and commercialization of newer, faster, and cheaper kits and protocols for diverse sequencing platforms might be the key factors reinforcing better SARS-CoV-2 genomic surveillance (8). Based on this, we expect each country to become more efficient in 2021. Therefore, this study evaluates the genomic surveillance efforts of all countries in 2021 compared to 2020. Most countries are indeed doing more SARS-CoV-2 sequencing and submitting to GISAID faster in 2021; however, these improvements may be inadequate given the increase in the number of COVID-19 cases. Surprisingly, several countries have even decreased their sequencing efficiency relative to the number of infections. Nevertheless, one noteworthy change that we witnessed in 2021 is that most developing countries across all continents have improved their genomic surveillance significantly, and based on this, they have been able to contain the COVID-19 pandemic to some extent (5, 6, 9). In summary, this study recommends that all countries must increase their sequencing efficiency, i.e., the sequenced genomes per 1,000 cases, and submit these samples to open access platforms such as GISAID rapidly to keep up with the emergence of newer variants of SARS-CoV-2.

## Author Contributions

GSh conceived the idea. UM and GSh designed the experiments. All authors wrote and edited the manuscript.

## Funding

UM acknowledges INSPIRE Ph.D. Fellowship support from the Department of Science and Technology (DST), Government of India. GSh was supported by the DST-INSPIRE Faculty Award from DST, Government of India. In addition, this work was partially supported by the Department of Electronics, IT, BT, and S&T of the Government of Karnataka, India.

## Author Disclaimer

The views expressed in this letter are those of the authors and not necessarily those of either funding agency or any other institution.

## Conflict of Interest

The authors declare that the research was conducted in the absence of any commercial or financial relationships that could be construed as a potential conflict of interest.

## Publisher's Note

All claims expressed in this article are solely those of the authors and do not necessarily represent those of their affiliated organizations, or those of the publisher, the editors and the reviewers. Any product that may be evaluated in this article, or claim that may be made by its manufacturer, is not guaranteed or endorsed by the publisher.

## Supplementary Material

Supplementary Table S1 Country-wise comparative distribution: The table lists CSTlag efficiency, sequencing, case increase, and sequencing efficiency for all 208 countries in 2020, 2021, and a comparison of 2021 vs. 2020. Each category is shaded in different color codes, and their fold-change information is also shaded in the same color. Values with less than one-fold change or negative % change are in red fonts. Countries that have submitted more than 1000 genomes are shaded yellow in Column A. Countries with less than 1,000 genomes are shaded gray in columns H, I, and J.

Supplementary Table S2 Continent-wise statistics. The table lists CSTlag efficiency, sequencing, case increase, and sequencing efficiency for six continents in 2020, in 2021, and a comparison of 2021 vs. 2020.

## References

[1] Zhu N Zhang D Wang W Li X Yang B Song J. A novel coronavirus from patients with pneumonia in China, 2019. N Engl J Med. (2020) 382:727–33. 10.1056/NEJMoa2001017 31978945 PMC7092803

[2] Li J Lai S Gao GF Shi W. The emergence, genomic diversity and global spread of SARS-CoV-2. Nat. (2021) 600:408–18. 10.1038/s41586-021-04188-6 34880490

[3] Kamil JP. Virus variants: GISAID policies incentivize surveillance in global south. Nature. (2021) 593:341. 10.1038/d41586-021-01310-6 34007076

[4] Kalia K Saberwal G Sharma G. The lag in SARS-CoV-2 genome submissions to GISAID. Nat Biotechnol. (2021) 39:1058–60. 10.1038/s41587-021-01040-0 34376850

[5] Adepoju P. Genomic surveillance for COVID-19 in West Africa. Nat Africa. (2021). 10.1038/d44148-021-00126-w

[6] Adepoju P. African Coronavirus Surveillance Network Provides Early Warning for World. (2022). Available online at: https://www.nature.com/articles/d41587-022-00003-3 (accessed January 27, 2022).

[7] Editorial. The viral arms race. Nat Biotechnol. (2021) 39:527. 10.1038/s41587-021-00937-0 33948002 PMC8095470

[8] Oude Munnink BB Worp N Nieuwenhuijse DF Sikkema RS Haagmans B Fouchier RAM. The next phase of SARS-CoV-2 surveillance: real-time molecular epidemiology. Nat Med. (2021) 27:1518–24. 10.1038/s41591-021-01472-w 34504335

[9] Mallapaty S. India's neighbours race to sequence genomes as COVID surges. Nature. (2021) 593:485–6. 10.1038/d41586-021-01287-2 33986507

